# Living without Oxygen: Anoxia-Responsive Gene Expression and Regulation

**DOI:** 10.2174/138920209787847032

**Published:** 2009-04

**Authors:** Kevin Larade, Kenneth B Storey

**Affiliations:** Institute of Biochemistry and Department of Biology, Carleton University, 1125 Colonel By Drive, Ottawa, Ontario, K1S 5B6, Canada

**Keywords:** Anoxia-responsive gene expression, cDNA library screening, anaerobiosis, mollusk, *Littorina littorea*, metabolic rate depression.

## Abstract

Many species of marine mollusks demonstrate exceptional capacities for long term survival without oxygen. Analysis of gene expression under anoxic conditions, including the subsequent translational responses, allows examination of the functional mechanisms that support and regulate natural anaerobiosis and permit noninjurious transitions between aerobic and anoxic states. Identification of stress-specific gene expression can provide important insights into the metabolic adaptations that are needed for anoxia tolerance, with potential applications to anoxia-intolerant systems. Various methods are available to do this, including high throughput microarray screening and construction and screening of cDNA libraries. Anoxia-responsive genes have been identified in mollusks; some have known functions in other organisms but were not previously linked with anoxia survival. In other cases, completely novel anoxia-responsive genes have been discovered, some that show known motifs or domains that hint at function. Selected genes are expressed at different times over an anoxia-recovery time course with their transcription and translation being actively regulated to ensure protein expression at the optimal time. An examination of transcript status over the course of anoxia exposure and subsequent aerobic recovery identifies genes, and the proteins that they encode, that enhance cell survival under oxygen-limited conditions. Analysis of data generated from non-mainstream model systems allows for insight into the response by cells to anoxia stress.

## INTRODUCTION

Humans view oxygen as essential for life but many organisms are actually excellent facultative anaerobes, able to take advantage of the high ATP yield from oxidative phosphorylation when oxygen is available but equally able to rely on fermentation reactions to generate energy when oxygen is lacking. Indeed, many invertebrate species demonstrate a remarkable capacity for long-term survival without oxygen. Anoxia tolerance has been particularly well-studied in species of marine mollusks that populate the littoral (or intertidal) region [1, 2]. Oxygen levels in this zone vary naturally as a result of a number of factors; for example, agitation of bottom sediment and silt, presence of toxins in the water, and high rates of respiration (by both plants and animals) in small tide pools can all deplete oxygen supply in the water. Life in the intertidal zone is further challenging because, in addition to the factors listed above, there is a cyclic availability of oxygenated water; at high tide, animals are submerged and can breathe with their gills whereas at low tide, animals are frequently exposed to air and unable to take up oxygen. For all of these reasons, most intertidal invertebrates have very well developed capacities for long term survival without oxygen (i.e. anaerobiosis) [3-5]. This review focuses on recent studies that have characterized the molecular biology of anoxia tolerance in marine mollusks, particularly extensive work with the periwinkle snail, *Littorina littorea*. Analysis of gene expression during anoxia, including the subsequent translational responses, allows examination of the functional mechanisms that support and regulate natural anaerobiosis and gives us a better understanding of how many organisms on earth can make reversible transitions to life without oxygen.

## METABOLIC SUPPRESSION

A recent review of the metabolic responses to anoxia in marine mollusks provides considerable detail of the biochemical mechanisms involved [3]. The underlying theme revolves around energy balance, particularly the balance between consumption and conservation of ATP. In a typical mollusk under aerobic conditions, lipids, carbohydrates or amino acids can all be used as fuels for respiration. When oxygen becomes limiting, however, there is a shift to carbohydrate catabolism as the primary fuel for ATP generation. Oxidation of hexose phosphates (derived from glucose or glycogen) *via *glycolysis produces ATP in substrate-level phosphorylation events. The ATP yield is low compared with that available from the complete oxidation of carbohydrates by the tricarboxylic acid cycle, but anoxia tolerant mollusks exploit and enhance this fermentative pathway to sustain survival. A number of biochemical mechanisms can be adjusted to support facultative anaerobiosis in mollusks [6]. These include maintenance of large tissue stores of fermentable fuels (primarily glycogen but also some amino acids), coupling additional substrate-level phosphorylation reactions to glycolysis to increase ATP output, and producing less acidic, alternative end products to lactate (e.g. succinate, propionate, acetate). These adaptations allow mollusks to maximize anoxic survival time based on a fixed reserve of internal fuels (review in greater detail in [3]).

Research by our lab and others determined that metabolic rate suppression, particularly tight control over macromolecular synthesis, is key to long-term survival without oxygen [3, 7-9]. Metabolic rate, a measure of net ATP turnover, approaches a level that matches the anoxic rate of ATP production, allowing mollusks to maintain long-term homeostasis. Energy-consuming processes, particularly pathways of macromolecule biosynthesis, respond to fluctuation in ATP supply, making them an obvious target for suppression during molluskan anaerobiosis. Stress-induced suppression of macromolecular synthesis has been documented in several invertebrate systems [10-12], and comprehensively studied in the anoxia-tolerant marine gastropod mollusk, *L. littorea* [3]. Short- and long-term anoxia exposure resulted in a dramatic reduction in transcription (42-50% decrease, relative to normoxic controls), with the rate of mRNA elongation reduced 68% relative to the normoxic rate [7]. In parallel, a strong reduction in protein synthesis (49% of normoxic values) was sustained over 48 h of anoxia exposure, with a rapid return to near control rates (86% of normoxic values) when oxygen was reintroduced [8]. Both gene expression and subsequent protein synthesis are energy expensive processes that require a supply of ATP, substrates (including nucleotides and amino acids), and the assembly machinery for transcription and translation. For this reason, resources are conserved during anaerobiosis by scaling back the transcription and translation of most genes. However, also key to survival is the up-regulation of a small suite of genes whose protein products play a critical role in cell preservation, facilitating both long-term viability under low oxygen and subsequent recovery when aerobic conditions return.

## GENETIC RESPONSE

Gene screening allows for identification of genes that may not have been previously associated with a particular stress, but perform a function that can be directly related to increased survival. Our laboratory and others have described stress-induced up-regulation of several genes in marine mollusks [13-24]. A variety of methods and techniques are available to investigators to identify stress-specific gene expression and in many cases, such genes may reveal additional unknown metabolic responses and/or adaptations to the stress.

Current technology offers the opportunity to examine the expression profiles of hundreds of genes, allowing researchers to probe multiple gene families and a variety of cellular functions simultaneously. Various screening tools are commercially available for several of the major animal model systems (e.g. human, rat, *Drosophila*, *Caenorhabditis elegans*). With appropriate attention to positive and negative controls, these can be used cost-effectively for studies with animal species that are outside the mainstream. High-throughput screening technology can be exploited with mollusk systems to examine the responses of large numbers of identified genes. Even though overall cross-reactivity may be low, many genes contain highly conserved domains and motifs and can therefore be evaluated with a heterologous screening approach. Successful cross-hybridization (using either cDNA based screening or antibody detection) that provides a reproducible response produces the initial leads that can be confirmed later using standard techniques such as Northern or Western blotting. Microarrays are an excellent tool for screening a large population of mRNA transcripts with relative ease. The percentage of genes that crossreact declines with phylogenetic distance, but screening provides hundreds of hits for follow-up studies.

Our lab used 19K human arrays to search for anoxia-responsive gene expression in *L. littorea* hepatopancreas (the liver-like organ of mollusks) [3]. Use of such cDNA arrays for screening phylogenetically “distant” organisms such as *L. littorea* does not successfully survey all of the thousands of genes represented on the array, since sequence identity between many mammalian and molluskan gene homologues is low. This is an obvious limitation of heterologous probing. However, even a low percentage of cross-reactivity still provides an assessment of the response to anoxia by hundreds of known genes and sequenced ESTs. Indeed, as expected, our study with *L. littorea *found that cross-reactivity was low (just 18.35%), and of these, most genes showed no significant change in transcript levels (88.8%) or reduced transcript levels (0.6%) following anoxia exposure, as compared with aerobic controls. This finding is consistent with the suggestion that most transcripts are maintained or sequestered during anoxia, readily available for when oxygen is reintroduced and normal translational activity is resumed, a concept that is further explored in Section 4. However, the key result from the array screening was the identification of many genes that were apparently up-regulated in response to anoxia; although these were only 10.6% of the crossreacting genes on the array, this represented in excess of 300 genes. These genes fell into a number of classes including selected protein phosphatases and protein kinases, mitogen-activated protein kinase-interacting factors, translation factors, antioxidant enzymes, iron-binding proteins and nuclear receptors (Larade and Storey, unpublished data). More recently, a number of researchers have developed species-specific microarrays for selected marine mollusks that will prove useful for future studies, particularly for species that are important to the aquaculture industry. These include a novel low-density oligonucleotide microarray, consisting of 24 mussel genes [25]; an oyster cDNA microarray containing 4460 sequences derived from *Crassostrea virginica* and 2320 from *Crassostrea gigas *produced by an international group of collaborators [26]; and a cDNA microarray containing 1714 probes constructed from the mussel *Mytilus galloprovincialis *[27]. In addition, a functional genomics initiative known as the Marine Genomics project has prepared and analyzed microarray data for 19 species databases (over 46,000 EST sequences) [28]. By enabling cross-species data comparison and data mapping, users can refine and share their research within a marine-focused environment.

The data provided from our microarray analysis of *L. littorea *generated a number of leads for further characterization and, very importantly, identified up-regulated genes whose protein products have never before been associated with anoxia survival. Thus, significant additions to our knowledge of the genes, proteins, and cell functions that contribute to anoxia tolerance were made. However, as mentioned above, the caveat to heterologous screening is that candidate genes must always be confirmed as anoxia-responsive by one or more additional methods such as RT-PCR or Northern blotting as well as, when possible, Western blotting to look at protein expression or some form of functional assay such as measuring enzyme activity. Furthermore, nucleotide sequencing of a partial or full mollusk sequence followed by comparisons with sequence banks is important for confirming identity. Such findings have expanded the landscape of known genetic responses that play a significant role in anoxia survival, as well as provided multiple new avenues of research to pursue.

Although gene screening using DNA arrays in a homologous manner has become the preferred method recently for analyzing organismal response to stress, investigators working with a wide range of vertebrate and invertebrate model animals do not currently have this resource and, furthermore, heterologous screening can only assess those genes that crossreact with the sequences on the array. To circumvent such limitations, the established technique of cDNA library screening can be used for the detection of differentially-expressed genes, a procedure that has been described in detail previously [18]. Construction and screening of a cDNA library is costly and laborious and favors the discovery of mRNA species that are in abundant supply, but it has the key benefit of being able to find novel, species-specific transcripts. Using a cDNA library prepared from *L. littorea*, we have identified a number of such genes in periwinkle hepatopancreas [14, 15, 18] that perform potentially novel functions under hypoxic or anoxic conditions. Although transcripts of each of the up-regulated genes have been shown to accumulate under anoxia, their patterns of expression show considerable variability. Some genes are actively transcribed and translated during the anoxic period itself [14]. These we hypothesize are necessary for anoxia survival, since the ATP expenditure associated with their transcription should not otherwise be tolerated under anoxic conditions when energy conservation is paramount (as described in Section 2). In other cases, transcripts may contain a conserved sequence that allows binding of specific proteins at the 3’ or 5’ end of the transcript to protect them from degradation. Our lab has identified the heavy chain of ferritin [16] as a member of this class in periwinkles, as discussed in the next paragraph. Other transcripts contain an internal ribosome entry signal (IRES) that can allow for selective translation of stress-responsive proteins under conditions where global translation is strongly suppressed. An example of this type of gene is ATF4/cyclic AMP (cAMP)-responsive element binding protein 2 [29], which regulates the integrated stress response (ISR). The ISR is a signaling pathway triggered through phosphorylation of the eurkaryotic ribosomal Initiation Factor 2 alpha (eIF2α), a pathway explored further in Section 4. Gene transcripts that accumulate in response to anoxia, but are not immediately translated, are also important. Transcripts of such genes are protected from degradation to preserve them for translation when needed, such as during the transition back from anoxic to aerobic life [17]. These transcripts may contain conserved sequences that allow binding of specific proteins to protect them from degradation, as described above, or transcripts may attract proteins that act as a signal or tether, promoting them to be sequestered into protective granules. As reviewed by Rzymski and Harris [30], stalled transcripts accumulate in aggregates known as stress granules following anoxic challenge. Such granules, consisting of halted pre-initiation complexes containing components such as mRNA, small ribosomal subunits, ribosomal initiation factors (e.g. eIF3, eIF4E, eIF4G), and poly(A) binding protein (PABP), have been proposed to protect mRNA until rapid re-initiation of protein synthesis can resume following removal of the stress. The mechanism for mRNA transcript inclusion or exclusion into stress granules following onset of a particular stress remains to be determined [31]. Involvement of a variety of proteins that can bind to specific regions within an mRNA transcript has been proposed, including PABP-1, TIA-1, TIAR and others [32, 33]. Although many mRNAs are actively recruited to stress granules, others are shuttled into degradation pathways [34] or stabilization [35]. Exclusion of stabilized mRNAs from stress granules may be indicative of a critical role that their protein products can play in dealing with the stress; hence, stabilized transcripts can be retained in the polysome fraction and translated during stress exposure. These often play an active role during the stress period, such as transcripts encoding heat-shock protein 70 (HSP70) [36], heat-shock protein 90 (HSP90) [37], and ferritin heavy chain [16]. An examination of transcript status over the course of anoxia and recovery periods provides insight into which genes, and the proteins that they encode, enhance cell survival under oxygen-limited conditions.

The roles of various anoxia-responsive genes that have been identified as up-regulated in *L. littorea via *different screening methods can be inferred based on their known functions in other organisms. For example, we found that transcripts of ferritin heavy chain accumulated during anoxic exposure in snail tissues, with a subsequent increase in ferritin heavy chain protein [16]. Ferritin is a huge protein consisting of 24 heavy and light subunits that surround a core of up to 4500 iron atoms [38]; its function is to sequester iron and prevent the metal from catalyzing nonenzymatic reactions that generate damaging reactive oxygen species (ROS). Up-regulation of ferritin as well as the transferrin receptor protein that is needed for iron uptake into cells has now been demonstrated in several models of hypoxia/anoxia tolerance [39]. It is well known that ferritin transcripts contain cis-acting nucleotide sequences in the 5’-UTR called iron regulatory elements (IREs) that are recognized by cytosolic RNA-binding iron-regulatory proteins (IRPs). IRPs bind and prevent the transcript from associating with ribosomes (blocking translation), except when iron levels rise, in which case an interaction between iron and IRP causes a dissociation of IRP from ferritin transcripts, triggering their translation [40]. Oxygen is one of several signals that regulates this system; hypoxia exposure reduces the RNA binding activity of IRPs, an effect reversed by reoxygenation [41]. Anoxia exposure of snails may reduce the number of IRP-blocked ferritin transcripts promoting translation of ferritin mRNA, a premise supported by the observation that ferritin mRNA transcripts remain associated with polysomes during anoxia [16]. The up-regulation of iron storage proteins under anoxic conditions strongly suggests that long-term viability is promoted by mechanisms that minimize iron-mediated ROS generation by maximizing the uptake and storage of iron in hypometabolic states. Although oxygen levels are low under hypoxic/anoxic conditions, there is a critical need for iron sequestering for two reasons: (a) to minimize free Fe^2+^ so as to limit the potential for generating ROS, particularly when oxygen is rapidly reintroduced into tissues during the recovery period, or (b) to store excess iron during a time when the net rate of biosynthesis of iron-containing proteins is low. The first reason is particularly attractive given that anoxia tolerant species also typically show strong anoxia-induced up-regulation of genes for a variety of antioxidant proteins/enzymes [42, 43].

Our lab has also used cDNA library screening to discover a number of novel genes that have conserved domains and motifs, but have yet to be identified in current genome projects or by sequence searches in NCBI databases. Such genes may be unique to the molluskan (or invertebrate) stress response. This is not surprising since the vast majority of molluskan genomes remain uncharacterized to date. These genes may encode unique proteins that are required for tolerance and survival of anoxic conditions, genes that may not be found in the genomes of anoxia-intolerant species.

Some of the novel genes that are anoxia-responsive in *L. littorea, *but cannot yet be identified in databases, include *kvn* [14] which contains a number of common protein motifs; *sarp-19* [15] which contains an uncommon, yet well-characterized, motif with a known function; and *sarp-3* (detailed below for the first time), which encodes a novel gene with significant sequence homology across the animal kingdom. For this type of gene that appears to be present across phylogeny, further characterization and potential identification can make use of both mammalian and invertebrate (e.g. *Drosophila*, *C. elegans*) model systems, both of which have a large number of tools available to characterize the role that such a gene may play. These can include an examination of the promoter [44], performing a gene knock-out and establishing a phenotype [45, 46], *in vitro* analysis using cell lines with endogenous and exogenous treatments [47], and analysis of a potential mechanism [48]. To date, none of these studies have been undertaken on the mammalian version of the *sarp-3* gene.

The anoxia-responsive novel gene *kvn* from *L. littorea *extends 525 bp and contains a full open reading frame encoding 99 residues. The predicted molecular mass of KVN protein is 12 kDa and it contains an N-terminal hydrophobic signal sequence, likely targeting the protein to the endoplasmic reticulum for processing and secretion. Spacing of the several cysteine clusters in the protein suggest that KVN may be an iron–sulfur protein that binds iron and is related to the ferredoxin family [14]. A possible role for KVN might be in mediating electron transfer reactions during anoxia or recovery, perhaps in mitochondrial metabolism, by directing electron flow into substrate level phosphorylations associated with metabolite synthesis. The novel gene *sarp-19* (snail anoxia-responsive protein, 19 kDa) contains an open reading frame encoding a 168 amino acids with an N-terminal signal sequence and two EF-hand domains [15]. The known function of EF-hand domains is calcium binding, which induces a conformational change causing activation (or inactivation) of target proteins [49]. The function of SARP-19 in anaerobiosis may include calcium-activated signaling or calcium sequestering, which may have a key physiological function in extracellular spaces of marine mollusks. The shift from aerobic to anaerobic life is accompanied by regulated dissolution of the calcium carbonate shell, with released bicarbonate acting as a buffer against the build up of acidic end products of fermentative metabolism [50]. Aerial exposure stimulates mollusks to ‘seal’ themselves within their shells, becoming a closed systems that aids water and osmotic balance but results in elevated hemolymph calcium levels during anaerobiosis [51]. Since calcium is a key signaling molecule, with intracellular levels regulated at very low values by sarcoplasmic reticulum and plasma membrane Ca^2+^ pumps, elevated production of a Ca^2+^-binding protein under anoxia would help minimize free Ca^2+^ levels.

Another newly discovered anoxia-responsive transcript retrieved from the *L. littorea* hepatopancreas cDNA library has been named snail anoxia-responsive protein-3 (*sarp-3*). Our investigation of this transcript provides a good example of the many types of analyses that can be used to gain information about the structure, function and expression of novel genes. The isolated clone was a 911-bp cDNA containing a 567-bp open reading frame (ending with a stop codon), absent the 5’-end of the sequence; this coded for a putative protein of 188 amino acids. The clone was subsequently used to produce a specific cDNA probe to detect *sarp-3* on Northern blots. Total RNA prepared from hepatopancreas samples was denatured and separated on formaldehyde agarose gels and probed with radiolabeled *sarp-3*. A single band was detected at ~1.2 kb. Advances in the field of functional genomics and bioinformatics, combined with the availability of a database of annotated genes, have spawned a wealth of software and analysis programs that allow researchers to examine nucleotide (and protein) sequences. Differentially expressed mRNAs are sequenced and compared to existing and annotated sequences; novel or unknown genes often have no definitive match, although some conserved domains and/or motifs may be identified and provide an abbreviated sequence match. When a gene is highly conserved across several phyla, it is likely that the gene has been maintained as a result of selective pressure, with such genes performing a valuable or required function. A homology search in t-BLASTx (a search of translated nucleotide databases using a translated nucleotide query) revealed homologues of SARP-3 protein in a wide range of organisms, in particular with high conservation within a 50 amino acid segment running from residues 21-70, as shown in Fig. (**1**).

**Fig. (1):**
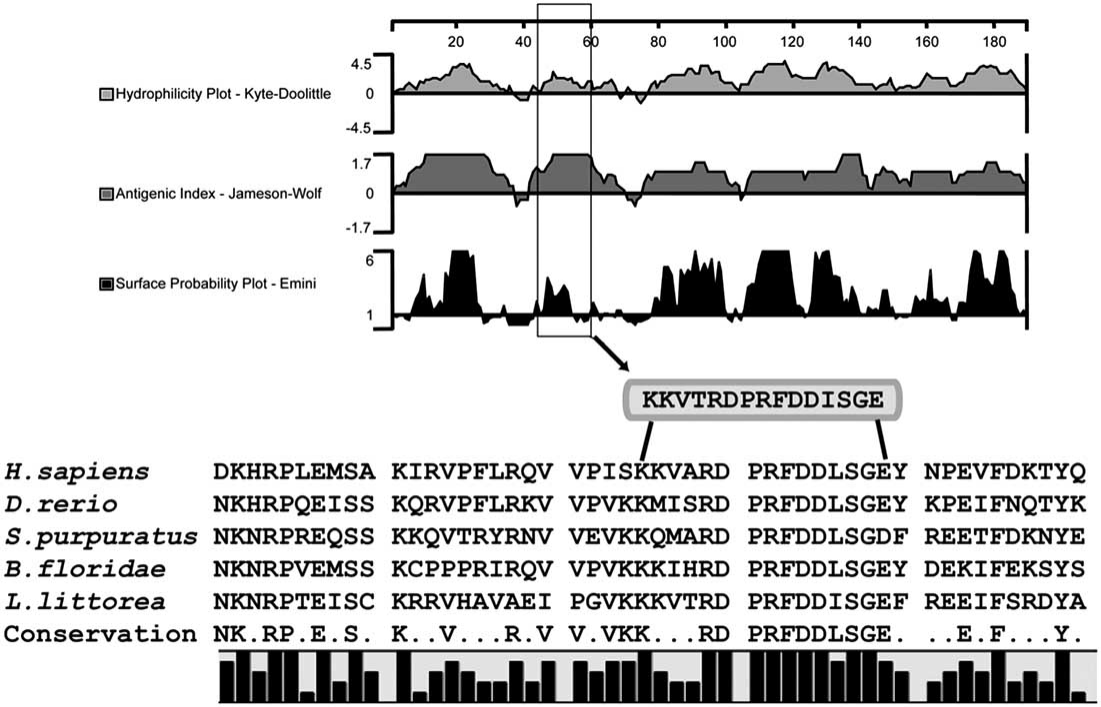
Analysis of the SARP-3 amino acid sequence to identify antigenic domains for epitope selection. The primary SARP-3 sequence containing 188 amino acids was analyzed using three different algorithms (DNA Star protein analysis software, Protean 4.0), including a hydrophilicity plot (Kyte/Doolittle), antigenic index (Jameson/Wolf), and surface probability plot (Emini), to identify antigenic determinants in SARP-3 (top panel). A multiple alignment of SARP-3 homologues (identified using t-BLASTx), was performed to identify regions of conservation (bottom panel) and the selected antigenic sequence, representing amino acid residues 45-59 in SARP-3, is identified by a box. Sequence source are (with accession numbers): *Strongylocentrotus purpuratus* (XM_001191687); *Branchiostoma floridae* (XM_00223-8688); *Homo sapiens* (NM_033112.2); *Danio rerio* (NM_001079944); *Littorina littorea* (FJ664185).

Determining the function of novel genes is a challenge currently facing many of the large genome projects. An mRNA sequence contains much information, but it is the functioning protein that performs the important physiological task. In order to anticipate the function of a particular gene product, the gene sequence is usually translated into a predicted protein sequence, which can then be analyzed for structural homology and conserved regions (known as domains or motifs) that have been identified in other proteins as having known functions. In addition, protein expression patterns, as well as identification of subcellular localization and interacting proteins, can be achieved with a suitable antibody. To determine the optimal epitope for antibody production, we performed a series of analyses on the SARP-3 amino acid sequence, determining hydrophilicity, antigenicity, and surface probability of the putative protein (Fig. **1**). Such procedures, when used together, are useful in developing synthetic peptide immunogens, particularly those that are surface-exposed and have a high level of conservation between various homologues. We were able to identify a region optimized for antigen use, which included a high degree of homology between the SARP-3 sequence homologues identified through NCBI BLAST analysis. A short peptide antibody, shown in Fig. (**1**), was produced and was used in protein expression analysis.

**Fig. (2):**
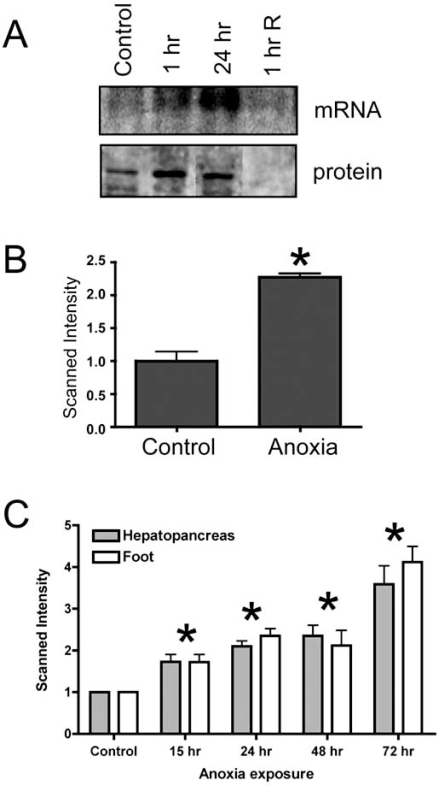
Expression analysis of *sarp*-3. (**A**) Sarp-3 mRNA and SARP-3 protein levels were determined in hepatopancreas of *L. littorea* given short term exposure *in vivo* to anoxia (1 or 24 h in N_2_ bubbled seawater) followed by 1 h of recovery in air-bubbled seawater. Total RNA was resolved on a 1.5% formaldehyde gel, blotted onto nitrocellulose, and hybridized at 45°C to ^32^P-labeled probe produced from the *sarp*-3 cDNA clone. A prominent band was detected at 1.2 kb. Blots were normalized by viewing the ethidium bromide (EtBr) stained gel under UV light and probing with 18S rRNA. Expression of SARP-3 protein is shown in Western blots, with a prominent band detected at ~40 kDa. Western blots were standardized by comparing the protein levels in each sample lane after the membrane was stained with a reversible Ponceau S stain. (**B**) Expression of *sarp*-3 transcripts in hepatopancreas explants exposed to anoxia *in vitro*. After incubation (15 h at 4°C in aerated vs N_2_-bubbled seawater), total RNA was isolated from the hepatopancreas samples, resolved on a 1.5% formaldehyde gel, blotted onto nitrocellulose, and hybridized to ^32^P-labeled *sarp*-3 probe. Histograms show the scanned intensity of experimental samples relative to normoxic controls; data are means ± SEM, *n* = 3 independent trials. (**C**) *Sarp*-3 mRNA expression was determined in hepatopancreas and foot muscle of *L. littorea* over a time course of up to 72 h of anoxic exposure *in vivo*. The histogram shows mean band intensities of *sarp*-3 mRNA (±SEM, *n* = 6-9) in anoxic samples relative to control levels. Statistical testing used analysis of variance followed by the Dunnett’s test; * - significantly different from normoxic controls P < 0.05.

Expression of SARP-3 in *L. littorea *hepatopancreas was assessed over a time course of experimental exposure of snails to anoxic conditions *in vivo *(seawater bubbled with N_2_ gas), as described previously [14]. Northern blotting showed enhanced levels of *sarp-3 *mRNA transcripts under anoxia and Western blotting showed a comparable increase in SARP-3 protein abundance (Fig. **2A**). Both mRNA and protein levels decreased again within 1 h after snails were returned to oxygenated conditions. Further experiments used isolated hepatopancreas slices to examine *sarp-3 *expression in response to 15 h anoxia exposure *in vitro.* RNA was extracted from tissue samples, and *sarp-3 *transcript levels were determined by Northern blotting. Fig. (**2B**) shows that levels of *sarp-3* transcripts increased by 2.3-fold in hepatopancreas slices *in vitro *under anoxic conditions. Finally, a detailed *in vivo *time course of up to 72 h anoxia exposure showed a progressive, near-linear accumulation of *sarp-3 *mRNA in two tissues of snails: hepatopancreas and foot muscle (Fig. **2C**). Transcript levels rose by about 4-fold in both tissues over the time course. Expression of *sarp-3* in both muscle and nonmuscle tissue suggests that the protein may have a generalized role in cellular anoxia tolerance in all tissues.

**Fig. (3):**
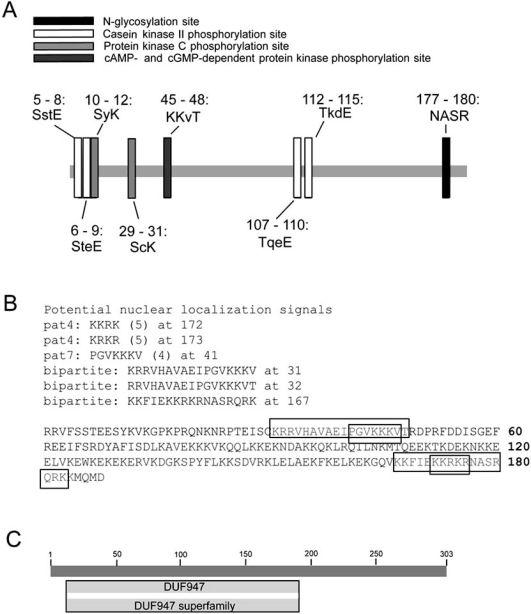
Identification of motifs and domains in SARP-3 protein. (**A**) Analysis of the primary sequence of SARP-3 identified a number of consensus sequences indicative of potential regions of post-translational modification. Putative sites included those for N-glycosylation, casein kinase II phosphorylation, protein kinase C phosphorylation, and cAMP-/cGMP-dependent protein kinase phosphorylation. (**B**) A variety of potential nuclear localization sequences were identified within the 188 amino acids of the SARP-3 protein that were retrieved. Both ‘pat 4’ and ‘pat 7’ targeting motifs were identified (continuous basic residues) as well as bipartite motifs (two regions of basic amino acids separated by non-conserved residues). (**C**) SARP-3 contains a conserved region (DUF947), characteristic of a family of eukaryotic proteins with unknown function. This domain is described in InterPro, a database of protein families (domains, regions etc.) in which identifiable features found in known proteins can be applied to new protein sequences (see INTERPRO: IPR009292) and Pfam, a database of protein families represented by multiple sequence alignments and hidden Markov models (see PFAM: PF06102). Both entries have been identified across a range of phyla.

The predicted protein sequence of SARP-3 was analyzed to search for conserved regions including domains or motifs, as well as localization sequences. Fig. (**3A**) shows that a number of isolated motifs were identified in SARP-3, including an N-glycosylation site, a cAMP-/cGMP-dependent protein kinase phosphorylation site, two putative casein kinase II phosphorylation sites, and four potential protein kinase C phosphorylation sites. In addition, potential nuclear localization sequences were identified (Fig. **3B**), as well as an unknown superfamily domain (DUF947) (Fig. **3C**). Analysis of the homologous sequences, seen in Fig. (**1**), did not yield additional information that was not attainable from the *L. littorea* sequence. Other sequences used in the alignment included *S. purpuratus* (198 residues, pI = 10.69, 23.9 kDa), *B. floridae* (235 residues, pI = 10.68, 28.1 kDa), *H. sapiens* (259 residues, pI = 10.87, 29.8 kDa), and *D. rerio* (320 residues, pI = 9.99, 37.9 kDa). Using the *H. sapiens* sequence as a model, no signal peptides were apparent in the N-terminal end of the protein and it contained the same nuclear localization sequences, with no other apparent targeting sequences. It is also overwhelmingly predicted to be a basic, nuclear protein. The function of SARP-3 is unknown at present and confirmation of its role will require considerable additional work, using many of the tools outlined throughout this section. However, given the presence of many potential protein kinase phosphorylation sites as well as putative nuclear localization signals, it is quite possible that SARP-3 might prove to be an anoxia-responsive transcription factor, co-activator/co-repressor, or involved in the transcription factor binding complex.

## THE FUNCTIONAL RESPONSE

As outlined in Sections 2 and 3, a key to survival during anoxia exposure, as well as making a successful transition back to aerobic life when oxygen is reintroduced, is the suite of mRNA transcripts that accumulate during anoxia. The roles of the proteins encoded by anoxia-responsive genes can be speculated upon based on whether: i) the protein shares key domains, motifs, secondary structure or other attributes known to be characteristic of a specific protein or protein family, ii) mRNA transcripts accumulate due to active transcription or due to protection from degradation, iii) the transcript is translated to produce the protein at the onset, during, or following anoxia exposure, and iv) the protein has a defined role(s) in other systems. These concepts have been addressed with specific examples in Section 3, however, using the *L. littorea* model as an example, this section demonstrates how independent observations are found to be interrelated in the context of metabolic suppression, as outlined in Section 2.

Protein synthesis is a metabolically expensive process that is strongly suppressed in anoxia-tolerant organisms experiencing longer-term oxygen deprivation. The rate of translation is balanced with the cell’s ATP generating capacity. Two primary mechanisms of global protein synthesis control are (i) the state of ribosome assembly, and (ii) reversible phosphorylation of specific proteins/factors that have a key role in ribosomal initiation and elongation. It has been demonstrated that a variety of stresses, including heat shock, viral infection, oxidative stress, and ischemia, elicit a rapid arrest of translation, promoting polysome disassembly [31]. Interestingly, specific RNA transcripts are actively transcribed as anoxia exposure begins, presumably providing for the synthesis of specific protein products that enhance survival. Cap-independent translation can be initiated at internal ribosome entry sites (IRES), which are less inhibited by phosphorylation of eIF2α (as described below), promoting the selective translation of specific mRNAs in stressed cells [52]. As mentioned above, translation initiation is a key step in protein synthesis [53]. Recruitment of both small and large subunits results in an active ribosome, which scans along specific mRNAs until it identifies an initiation site. A ribosome is composed of four different types of ribosomal RNA and over eighty ribosomal proteins. Ribosomal protein gene expression and subsequent translation is meticulously coordinated and, as foreshadowed in Section 2, is responsive to a variety of physiological conditions. Hence, it was not unexpected to observe a response by specific ribosomal proteins during anoxia exposure in *L. littorea* [13] or in response to physiological insults in other systems [54-58]. We identified ribosomal protein L26 as an actively transcribed gene that accumulated during anoxia. Transcript levels of L26 steadily increased over the course of anoxia exposure, *via *active transcription, and remained high following aerobic recovery. Ribosomal protein L26 is located at the interface of the large and small subunit, specifically at the peptidyl transferase center [59, 60] and has been shown to interact with elongation factor-2 (EF-2) [61]. Hence, L26 is proposed to be involved in subunit interactions, perhaps as a stabilizing factor, based on the observation that it undergoes structural rearrangement as ribosomal subunits associate [62]. L26 has also been correlated with EF-2 binding to the 60S ribosomal subunit preceding translocation of peptidyl-tRNA from the A to the P site during peptide bond formation [61, 63] and appears to be a key component involved in the formation (and resulting function) of the intact ribosome.

During active translation, mRNA transcripts are able to retain several polysomes moving along them. By contrast, when a transcript is not translationally active, polysomes dissociate into monosomes and free ribosomal subunits. This allows the translational status in a cell to be established by the degree of ribosomal assembly, by assessing the relative numbers of polysomes and monosomes in control versus stressed states. In an aerobic environment, polysomes associate and accumulate along mRNA transcripts, correlating with active protein synthesis. Various kinases can act as gatekeepers for translation in response to stress signals, through phosphorylation of initiation and elongation factors, such as eIF2α [64]. Following this modification, active ribosomes ‘run off’ mRNA transcripts resulting in disassembly of the polysome complexes. Phosphorylation of eIF2α, which promotes the binding of the initiator tRNA that holds methionine, to the 40S ribosomal subunit is correlated with inhibition of protein synthesis in *L. littorea*. In response to anoxia exposure, the content of phosphorylated eIF2α increased by >14-fold as compared with aerobic controls [7, 8]. This response was rapidly reversed following aerobic recovery. Ribosome distribution was also examined in *L. littorea* by separating monosomes and polysomes on a sucrose density gradient. A high proportion of ribosomes prepared from normoxic hepatopancreas were present as polysomes, consistent with active translation [8]. However, during anoxia exposure, most ribosomes were detected in the monosome fraction, indicating decreased activity of the protein synthesizing machinery. This effect was reversed upon re-oxygenation.

Suppression of protein synthesis (see Section 2), combined with phosphorylation of eIF2α and decreased polysome assembly, is indicative of blocked initiation of translation. This phenomenon was observed in *L. littorea* and is consistent with the observation that regulation of protein synthesis is primarily at the level of initiation for systems where the rate of protein synthesis is down- and up-regulated in a global manner [65]. Since the metabolic suppression described above is reversed during re-oxygenation, metabolism of organisms in the littoral zone surges. As outlined above, an abundance of L26 transcripts is in position to be translated when aerobic conditions return, providing a rapid increase in the capacity of the translational apparatus, as well as potentially improving ribosomal stabilization, allowing the system to cope with a demand for the increase in protein synthesis that occurs during aerobic recovery.

## SUMMARY

Depending on the particular function of the gene product, various genes may be transcribed at different times in response to anoxia stress. Early response genes are actively transcribed as oxygen tension decreases (during the hypoxic transition period but prior to full metabolic rate suppression). Other gene products are up-regulated to support long term survival during sustained anaerobiosis, resulting either from enhanced transcription or protection against degradation. Such gene products may also be involved in cell protection during aerobic recovery following reperfusion of oxygen (such as to provide defense against surges in ROS production). Gene products that accumulate during anoxia exposure, particularly those that result from active transcription or translation in the energy-restricted anoxic state, help the organism to survive without oxygen. Some mRNA transcripts even accumulate during the anoxic insult but are retained in translationally inactive pools, following the dissociation of active polysomes. Such transcripts are often protected or stored in anticipation of the inevitable return of oxygen into the system; hence, they likely play a key role during the re-oxygenation phase.

Analysis of data generated from the extensive examination of this marine gastropod model system allows for broad and specific insights into the response by cells to anoxia stress. The benefit of using a system such as *L. littorea* is that these mollusks are capable of enduring long periods of low oxygen tension. Previously characterized genes may demonstrate new or novel functions and can be incorporated into already existing schemas. Complicating this approach is the identification of novel ‘factors’, including genes and protein products, through methods such as cDNA library screening. Such new factors may have a role in existing pathways, as well as highlight potential new metabolic functions that are as yet undefined in their importance to anoxia survival.
